# Single-stage multilevel soft-tissue surgery in the lower limbs with spastic cerebral palsy: Experience from a rehabilitation unit

**DOI:** 10.4103/0019-5413.43395

**Published:** 2008

**Authors:** Anupam Gupta, Abhishek Srivastava, Arun B Taly, Thyloth Murali

**Affiliations:** Department of Psychiatric and Neurological Rehabilitation, National Institute of Mental Health and Neuro Sciences (NIMHANS), Bangalore, India; 1 Department of Neurology, National Institute of Mental Health and Neuro Sciences (NIMHANS), Bangalore, India

**Keywords:** Corrective surgery, deformity, spastic cerebral palsy

## Abstract

**Background:**

To assess the effect of single-stage multilevel soft-tissue surgery (Single Event Multiple Level Resections, SEMLR) on deformities and locomotion in patients with cerebral palsy (CP) with static contracture(s) in lower limbs.

**Patients and Methods:**

Study included 34 patients (M:F, 23:11) with mean age of 9.53 ± 3.92 years (4–16 years). Among them 22 had diplegia and four each had quadriplegia and right and left hemiplegia. Fourteen patients (41.2%) had their intelligence quotient (IQ) in the normal range (IQ ≥ 80), while others had mental retardation (MR) of varying severity: borderline MR (IQ = 70–79) in 12, mild MR (IQ = 50–69) in 5, and moderate MR (IQ = 35–49) in patients 3. All patients underwent surgery (total number of procedures 153, average 4.5 procedures/patient) over a period of 30 months (April 2005 to September 2007). Improvement in functional abilities and locomotion was assessed using Gross Motor Functional Classification Scale (GMFCS) scores and by physical examination.

**Results:**

Significant improvement in function was observed (*P* = 0.000) after surgery when comparing the preoperative and postoperative GMFCS scores. All patients were maintaining ambulation at a mean follow-up duration of 13.12 ± 6.07 months (3–24 months), with five patients using knee-ankle-foot orthoses (KAFO), 22 using ankle-foot orthoses (AFO), and six patients using knee gaiters. Sixteen patients were using walker, and two were using crutches as assistive devices.

**Conclusion:**

This study suggests that CP patients with good trunk control and static contractures at multiple joints in the lower limbs can be made ambulant with single-stage multilevel soft-tissue surgery. It has to be a team effort of the surgeon and the rehabilitation team in the postoperative period for the attainment of satisfactory goal.

## INTRODUCTION

Cerebral palsy (CP) is characterized by a disorder of movement and posture caused by nonprogressive injury to immature brain^1^ that results in change in muscle tone and posture, both at rest and with voluntary activity.^2^ The incidence is two to three per thousand live births,^3^ with a prevalence of 2.4 per thousand children^4^ and a slight male preponderance (M:F, 1.33:1).^5^ Spastic CP is the most common subtype, which constitutes about 70–80% patients of overall CP population.

Conservative (nonoperative) management of the spastic CP involving the lower limb includes physiotherapy in the form of stretching and range of motion exercise and use of special therapies, e.g., neurodevelopmental therapy, serial corrective cast, oral antispastic medications like baclofen, tizanidine, and diazepam and management of focal spasticity with use of injections of phenol/alcohol or botulinum toxin. In selected cases not responding to these treatments, selective dorsal rhizotomy^6^–^8^ and intrathecal baclofen^9^,^10^ have been tried.

These nonoperative methods may not be helpful in cases of static deformities in lower limbs with spastic CP and require soft tissue/bony corrective surgery, which not only corrects the static deformity but also helps in attaining upright posture, which reduces muscle and joint pain, restores muscle balance across the joint, and makes the limb amenable to donning of orthosis.

Multilevel surgeries or Single Event Multiple Level Resections (SEMLR) in the lower limb are performed to improve the gait of children with spastic CP.^11^ The treatment should be individualized and planned according to the patient's needs. Maintaining hygiene and independent and balanced sitting would be the primary goal in a child with total body involvement or quadriplegia. However, the primary aim in a diplegic child would be improvement of gait and adequate energy consumption.^12^

Different authors have recommended different specific age for SEMLR that varies from 4–8 years^13^ to just before the period of increased growth in adolescence.^14^ These studies suggest that children in whom surgical intervention is recommended are not a homogeneous group.

This retrospective study analyzed the data on 34 patients with spastic CP who were operated for lower limb deformities to attain improvement in personal hygiene, gait, and independent ambulation with assistive devices alone or with orthoses.

## MATERIALS AND METHODS

The present study included 34 patients (M:F, 23:11) with a mean age of 9.53 ± 3.92 years (4–16 years). All patients were admitted in the unit for corrective surgery from the neurorehabilitation outpatient services of the institute over a period of 30 months (April 2005 to September 2007). Informed consent was obtained from the parents. Twenty patients had diplegia and four each had quadriplegia and right and left hemiplegia. All children had attained good truncal balance, dynamic sitting, and good muscle power in both the upper limbs (at least grade 3 power in quadriplegics) or in unaffected limb (in hemiplegics). None of the patients had fixed spinal deformity or prior surgery for correction of lower limb deformity. Intelligence quotient (IQ) assessment was done for all children before surgery using Binet Kamat Tes' Battery or Vineland Social Maturation Scale (VSMS). Children with dyskinetic or mixed CP, those requiring bony surgeries, and those with severe or profound mental retardation (not trainable) were excluded from the study.

Static and dynamic conditions in the lower extremities were assessed with physical examination and Gross Motor Functional Classification Scale (GMFCS) scores.^15^ Unsupported sitting, standing, and gait properties were evaluated. Soft-tissue contractures in the lower limbs were assessed with special tests and joint examinations. Hip flexion deformity was assessed using Thomas Test and Prone-Rectus Test, and deformities were measured using goniometer according to the neutral 0 method. Hip abduction restriction in extension and in 90° of flexion was attributed to hip adductor muscle contracture. Hamstring tightness was assessed using popliteal angle and Phelps-Becker Test. Popliteal angle was measured with the patient in supine position and the hip in 90° of flexion. Equinus deformity at ankle was assessed using Silverskiold Test. Gait patterns such as crouching, scissoring, jump knee gait, toe-to toe gait, and toe-to-heel gait were noted in all the children clinically.

### Operative procedures

In static hip flexion deformities, psoas muscle release and rectus femoris release was carried out. In cases in which the deformity was minor and sartorius muscle and deep fascia were found tight, they were released. In case of adductor tightness, open myotomy of adductor longus and brevis was performed. In static knee flexion deformity, hamstring release was performed. Tenotomy of gracilis muscle along with lengthening of semitendinosus was performed. Semimembranous muscle was lengthened medially and biceps femoris laterally by aponeurosis recession or release. In all patients with static equinus deformities, Z-plasty was done for lengthening of Achilles tendon using Whites' Method.

### Postoperative protocol

After hip and knee surgeries, compression bandage and skin traction were applied to provide gentle stretching. After removal of sutures on the 10^th^ postoperative day, children were sent home with groin-to-toe cast in the operated limb(s). They were advised rest with cast. Patients were called after 4–6 weeks, when casts were removed and physiotherapy was started and measurement of orthoses was given simultaneously. Patients were trained to walk wearing orthoses with or without assistive devices.

In cases of surgery for equinus deformity only, above-knee cast was applied at the time of surgery with knee in 5–10° in flexion and ankle in about 5° dorsiflexion. Patients were advised rest (walking not permitted). After removal of sutures on 10^th^ postoperative day, children were discharged with cast and advised to come after 4–6 weeks. At follow-up, cast was cut in bivalved manner on the first day of admission, so that the lower end could be used as shell. Wax bath to both knee and ankle joints was started from the next day (to make joint supple), and passive range of motion started from 3^rd^ day both manually and with knee continuous passive motion (CPM) equipment. Articulated AFOs were prescribed and gait training with the orthosis and assistive device (walker or walking aluminum cane depending upon unilateral or bilateral surgery) was given. Same protocol was used in cases in which ankle deformity was operated along with hip and knee deformity.

### Statistical analysis

SPSS 15.0 was used for the analysis of the data. Descriptive analysis, frequency distribution was used to detect range, means, and standard deviations. GMFCS scores were compared preoperatively and postoperatively using nonparametric Wilcoxon Signed Rank Test.

### RESULTS

Preoperatively, all the children had static deformities in the lower limbs. All eight hemiplegic children and five out of 22 diplegics were ambulatory preoperatively albeit with deformities. The purpose of the soft-tissue surgery was to correct the deformity so that they can stand upright, don the orthoses, and can ambulate using it with or without assistive device for walking and improve personal hygiene in cases of adductor spastic contractures. Deformities in all the joints were recorded. There were 46 static equinus deformities, 20 static hip flexion deformities, four hip adductor deformities, and 83 hamstring static deformities (medial and lateral hamstrings) in 34 patients (68 legs). Mean hip flexion deformity was 26.36 ± 8.34°, mean popliteal angle was 98.12 ± 15.42°, and mean equinus deformity was 27.52 ± 9.38° preoperatively. Overall, 153 operative procedures were done in the patients with average of 4.5 procedures per child. All children attained complete or nearly complete correction of deformities after surgery.

Improvement in functional abilities and locomotion was assessed preoperatively and postoperatively using GMFCS scores and by physical examination. GMFCS was not applicable in nine patients in this study, as they were more than 12 years of age at the time of surgery. Preoperative and postoperative GMFCS scores of remaining 25 patients are mentioned in Table 1. There was significant improvement GMFCS score following surgery (*P* = 0.000). The level of mental retardation was assessed using test batteries mentioned earlier.[Table 2]

**Table 1:** Gross motor functional classification scale scores* in the preoperative and postoperative period

GMFCS score	Number of patients		Significance (*P* value)
		
	Preoperative	Postoperative	
Level I	–	–	0.000
Level II	–	8	
Level III	10	15	
Level IV	15	2	
Level V	–	–	

* Not applicable on 9 patients with age >12 years, GMFCS: Gross Motor Functional Classification Scale.

**Table 2:** Level of mental retardation among the patients in the study

Level of mental retardation	IQ	Number of patients	%
Borderline	70–79	12	35.3
Mild	50–69	5	14.7
Moderate	35–49	3	8.8
Severe	20–34	–	–
Profound	<20	–	–
Normal	≥80	14	41.2

No complications were noticed at hip (both for flexion and adductor sites) and knee at the operated site. Four children (four legs) with equinus deformity developed wound dehiscence at the suture line with loss of skin. Their length of stay was prolonged, antibiotics and daily wound dressing was done, and wounds healed with no further complications.

Two patients each with quadriplegia and hemiplegia had spastic (dynamic) contractures in wrist and finger flexors bilaterally and in the affected upper limb, respectively. Botulinum A toxin injections were given locally, followed by resting hand splints in the postoperative period. Both quadriplegic children were able to hold the walker for ambulation at the time of discharge.

Fourteen children with spastic diplegia were advised bilateral AFO for locomotion postoperatively. Out of these, 12 children also received bilateral knee gaiters. Two children with spastic diplegia and three with quadriplegia were advised bilateral KAFO for ambulation. All eight children with hemiplegia received AFO postoperatively. However, all four children with quadriplegia and 12 with diplegia required walker as assistive device. Two children each required axillary and elbow crutches as assistive device for walking.

Mean duration of follow-up in the study was 13.12 ± 6.07 months (3–24 months). Three children (8.82%) were therapeutic/exercise walker, 17 (50%) were ambulatory in the household/functional ambulators, and 14 children (41.17%) were community walker at the time of follow-up. Among the therapeutic walkers two had quadriplegia. All parents and children were satisfied with the results of surgery and reported improvement in functional abilities and locomotion in the follow-up. Their quality of life was better, and many of the children who were not at all able to stand before surgery were standing and walking with orthoses and assistive devices.

### DISCUSSION

Spastic CP is the most common subtype of all CP cases, and diplegia, hemiplegia, and quadriplegia are the most frequent clinical presentation, in that order in the spastic subtype. Conservative management in the form of physiotherapy, as soon as the diagnosis is made in infancy/early childhood, should be the first line of management.

Awareness among parents of these children is increasing in developing countries like India, especially in urban areas, and many cases are seen in the specialized tertiary centers at a very early age. However, there are still a significant proportion of CP cases that are brought only after the child is more than 3–5 years of age and has not started standing or walking.^16^ Apart from inability to walk, these children would already develop static/dynamic deformity across the various joints in the lower limb. Some of these ambulatory patients would be walking using the bamboo/hockey sticks or proper cane. When such nonambulatory cases with multiple static deformities in the lower limbs are brought first time to a tertiary care center from far off places, a single-stage multilevel corrective surgery along with physiotherapy may be more pragmatic than planning multistage surgical procedures with rehabilitation. It improves the treatment outcome of patients with spastic CP^17^. This is also logical because most of these patients, once ambulant after surgery, would not report for follow-up.

As complete recovery in spastic CP is impossible because of the brain lesion, surgical interventions are performed to provide, maintain, or improve musculoskeletal system function and to significantly improve the quality of life of these patients.^12^ Corrective surgery is required when the contractures are static and severe enough to interfere with movements and locomotion.^11^ The functional status of the child before surgery is an indication of the biomechanical disturbances within the limbs and joints and the child's overall neurophysiological condition. By restoring muscular balance, surgical treatment influences the presence and extent of dynamic and static joint deformity and improves the child's general condition.^18^

Zwick *et al*., in a case-control study involving 20 patients with spastic diplegia, observed that patients who underwent single-stage multilevel surgery walked faster with increased stride length and considerably increased knee joint range of motion when compared with the control group. These patients had improved knee extension during the stance phase of gait, which caused improved stance limb stability and facilitate an unhindered swing phase of the opposite limb after surgery.^19^

Molenaers *et al*., in another case-control series of 52 patients with spastic CP, compared the results of single-stage multilevel injections of botulinum toxin A with that of the surgery. They concluded that both these treatment modalities should be regarded as complementary rather than mutually exclusive, with both calling for an integrated approach.^20^

Consideration of age while planning corrective surgeries in spastic CP patients is very important. As cases with spastic diplegia and quadriplegia do not attain stable gait pattern before the age of 3–4 years, surgery is not advisable before the age of 4 years.^13^ In our study also, no patient was younger than 4 years of age at the time of surgery.

Earlier studies^21^,^22^ have documented the complications like recurrence of equinus deformity and overlengthening of Achilles tendon (T-A lengthening) resulting in crouched or calcaneus gait following isolated Achilles tendon lengthening procedure in spastic CP with diplegia and quadriplegia. Similarly, there are studies documenting complications following hamstring lengthening such as recurrence of deformity,^23^ development of recurvatum of knee due to spastic rectus femoris, and increased forward tilt of pelvis in ambulatory patients.^24^ As most of the patients in our study had hamstring surgery at the same time along with Achilles tendon lengthening and as these patients also received knee gaitors or KAFOs after surgery, such complications were not observed till the time of follow-up. Our follow-up duration was short (13 months), and these children may develop complications later, thereby requiring constant supervision.

There are reports of deterioration of gait over a period of time following single-stage multilevel soft-tissue surgeries in patients with spastic CP.^11^,^25^,^26^ Gough *et al*.^11^ suggested that surgical intervention, which results in the stabilization of a child's gait pattern without change in the kinematics represents an improvement on the natural history in the short term. The authors believe that the long-term outcome is less promising, as patients developed deformities after attaining skeletal maturity, irrespective of surgical or conservative management.

Johnson *et al*., in a longitudinal study of patients with spastic diplegia, showed similar results with deterioration in gait over a mean of 32 months, irrespective of the age of the child or history of previous operative intervention. The gait of those children who already had surgical intervention continued to deteriorate at the same rate as those who had not had surgical intervention.^25^ Although we did not observe any deterioration of the gait during follow-up, these studies warrant cautious approach and long term follow-up of all operated cases.

Orthoses are frequently used to improve the gait and to correct or maintain the deformity in CP. Understanding of the biomechanics of the various joints of the lower limb during normal gait, the pathophysiology and pathomechanics of gait disruption in children with CP, and the biomechanical characteristics of various orthoses is imperative to prescribe appropriate splint. Design, indications, and cost should be considered when choosing an orthosis.^27^ Various splints prescribed in the study were in accordance to the needs of the patients with the aim of not only to maintain the correction achieved during the surgery but also to improve the gait characteristics such as velocity, endurance, and cadence. Patients maintained the improvement during the follow-up, they achieved in the postsurgery period and at the time of discharge.

Preoperative and postoperative (and the follow-up) GMFCS scores were used to assess the functional improvement in the patients after surgery. This is a commonly used and validated scale for functional assessment in CP.^28^ Twenty-three out of 25 (92%) patients (excluding nine patients in whom it was not applicable as they were >12 years of age) in our study showed at least one grade improvement in GMFCS scores after surgery, which was maintained till the time of last follow-up. Thus, there was significant functional improvement in all the patients after the surgery. Zörer *et al*.^12^ found similar results in the spastic CP cases after single-stage multilevel soft-tissue surgeries. Patients and their parents were satisfied with the results of surgery and were happy that their children have become functionally independent after surgery albeit using orthoses with or without assistive devices.

### Limitations of the study

The foremost limitations being the small sample size and a short follow-up period. Some of the complications like recurrence of deformities and deterioration of gait appearing years after corrective surgery could not be observed in the present study. Further, gait analysis, an objective method of observing the improvement in the muscles and gait, was not performed because of nonavailability of the equipment during that period.

## CONCLUSION

Single-stage multilevel soft-tissue surgery in the lower limb(s) in children with spastic CP and good trunk control yields good results for locomotion. In developing countries like India, where the patients would not turn up for multistage surgery and follow-up, this is a cost-effective and logical approach. Almost all children showed satisfactory results with minimal complications after corrective surgery, and their functional ability improved significantly.

## References

[1] Molnar GE (1987). Long-term treatment of spasticity in children with cerebral palsy. Int Disabil Stud.

[2] Naeye RL, Peters EC, Bartholomew M, Landis JR (1989). Origins of cerebral palsy. Am J Dis Child.

[3] Kiely M, Lubin RA, Kiely JL (1984). Descriptive epidemiology of cerebral palsy. Public Health Rev.

[4] Hirtz D, Thurman DJ, Gwinn-Hardy K, Mohamed M, Chaudhuri AR, Zalutsky R (2007). How common are the “common” neurologic disorders?. Neurology.

[5] Johnson A (2002). Prevalence and characteristics of children with cerebral palsy in Europe. Dev Med Child Neurol.

[6] Steinbok P (2007). Selective dorsal rhizotomy for spastic cerebral palsy: A review. Childs Nerv Syst.

[7] Farmer JP, Sabbagh AJ (2007). Selective dorsal rhizotomies in the treatment of spasticity related to cerebral palsy. Childs Nerv Syst.

[8] Park TS, Johnston JM (2006). Surgical techniques of selective dorsal rhizotomy for spastic cerebral palsy: Technical note. Neurosurg Focus.

[9] Kolaski K, Logan LR (2007). A review of the complications of intrathecal baclofen in patients with cerebral palsy. Neurorehabilitation.

[10] Hoving MA, Van Raak EP, Spincemaille GH, Palmans LJ, Sleypen FA, Vles JS (2007). Intrathecal baclofen in children with spastic cerebral palsy: A double-blind, randomized, placebo-controlled, dose-finding study. Dev Med Child Neurol.

[11] Gough M (2004). Short-term outcome of multilevel surgical intervention in spastic diplegic cerebral palsy compared with the natural history. Dev Med Child Neurol.

[12] Zorer G, Dogrul C, Albayrak M, Bagatur AE (2004). The results of single-stage multilevel muscle-tendon surgery in the lower extremities of patients with spastic cerebral palsy. Acta Orthop Traumatol Turc.

[13] Bleck EE Orthopaedic Management in Cerebral Palsy. Clinics in Developmental Medicine No 99/100.

[14] Patrick JH, Roberts AP, Cole GF (2001). Therapeutic choices in the locomotor management of the child with cerebral palsy-more luck than judgement?. Arch Dis Child.

[15] Palisano RJ, Hanna SE, Rosenbaum PL, Russell DJ, Walter SD, Wood EP (1997). Model of gross motor function for children with cerebral palsy. Dev Med Child Neurol.

[16] Khan MA (2007). Outcome of single-event multilevel surgery in untreated cerebral palsy in a developing country. J Bone Joint Surg Br.

[17] Kokavec M (2006). Long-term results of surgical treatment of patients suffering from cerebral palsy. Bratisl Lek Listy.

[18] Jozwiak M (2002). The clinical value of multilevel soft tissue release in the management of dynamic and static limb deformities in cerebral palsy children. Ortop Traumatol Rehabil.

[19] Zwick EB, Saraph V, Strobl W, Steinwender G (2001). Single event multilevel surgery to improve gait in diplegic cerebral palsy: A prospective controlled trial. Z Orthop Ihre Grenzgeb.

[20] Molenaers G, Desloovere K, De Cat J, Jonkers I, De Borre L, Pauwels P (2001). Single event multilevel botulinum toxin type A treatment and surgery: Similarities and differences. Eur J Neurol.

[21] Sutherland DH, Zilberfarb JL, Kaufman KR, Wyall MP, Chambers HG (1997). Psoas release at the pelvic brim in ambulatory patients with cerebral palsy: Operative technique and functional outcome. J Pediatr Orthop.

[22] Dietz FR, Albright JC, Dolan L (2006). Medium-term follow-up of Achilles tendon lengthening in the treatment of ankle equines in cerebral palsy. Iowa Orthop J.

[23] Dhawlikar SH, Root L, Mann RL (1992). Distal lengthening of the hamstrings in patients who have cerebral palsy: Long-term retrospective analysis. J Bone Joint Surg Am.

[24] Beals RK (2001). Treatment of knee contractures in cerebral palsy by hamstring lengthening, posterior capsulotomy and quadriceps mechanism shortening. Dev Med Child Neurol.

[25] Johnson DC, Damiano DL, Abel MF (1997). The evolution of gait in childhood and adolescent cerebral palsy. J Pediatr Orthop.

[26] Kadaba MP, Ramakrishnan HK, Wooten ME (1990). Measurement of lower extremity kinematics during level walking. J Orthop Res.

[27] Davids JR, Rowan F, Davis RB (2007). Indications for orthoses to improve gait in children with cerebral palsy. J Am Acad Orthop Surg.

[28] Wood E, Rosenbaum P (2000). The gross motor function classification system for cerebral palsy: A study of reliability and stability over time. Dev Med Child Neurol.

